# External validation: a simulation study to compare cross-validation versus holdout or external testing to assess the performance of clinical prediction models using PET data from DLBCL patients

**DOI:** 10.1186/s13550-022-00931-w

**Published:** 2022-09-11

**Authors:** Jakoba J. Eertink, Martijn W. Heymans, Gerben J. C. Zwezerijnen, Josée M. Zijlstra, Henrica C. W. de Vet, Ronald Boellaard

**Affiliations:** 1 Department of Hematology, Amsterdam UMC Location Vrije Universiteit Amsterdam, De Boelelaan 1117, 1081 HV Amsterdam, The Netherlands; 2 Imaging and Biomarkers, Cancer Center Amsterdam, Amsterdam, The Netherlands; 3 Epidemiology and Data Science, Amsterdam UMC Location Vrije Universiteit Amsterdam, Amsterdam, The Netherlands; 4 Methodology, Amsterdam Public Health Research Institute, Amsterdam, The Netherlands; 5 Radiology and Nuclear Medicine, Amsterdam UMC Location Vrije Universiteit Amsterdam, Amsterdam, The Netherlands

**Keywords:** Internal validation, External validation, Model performance, CV-AUC

## Abstract

**Aim:**

Clinical prediction models need to be validated. In this study, we used simulation data to compare various internal and external validation approaches to validate models.

**Methods:**

Data of 500 patients were simulated using distributions of metabolic tumor volume, standardized uptake value, the maximal distance between the largest lesion and another lesion, WHO performance status and age of 296 diffuse large B cell lymphoma patients. These data were used to predict progression after 2 years based on an existing logistic regression model. Using the simulated data, we applied cross-validation, bootstrapping and holdout (*n* = 100). We simulated new external datasets (*n* = 100, *n* = 200, *n* = 500) and simulated stage-specific external datasets (1), varied the cut-off for high-risk patients (2) and the false positive and false negative rates (3) and simulated a dataset with EARL2 characteristics (4). All internal and external simulations were repeated 100 times. Model performance was expressed as the cross-validated area under the curve (CV-AUC ± SD) and calibration slope.

**Results:**

The cross-validation (0.71 ± 0.06) and holdout (0.70 ± 0.07) resulted in comparable model performances, but the model had a higher uncertainty using a holdout set. Bootstrapping resulted in a CV-AUC of 0.67 ± 0.02. The calibration slope was comparable for these internal validation approaches. Increasing the size of the test set resulted in more precise CV-AUC estimates and smaller SD for the calibration slope. For test datasets with different stages, the CV-AUC increased as Ann Arbor stages increased. As expected, changing the cut-off for high risk and false positive- and negative rates influenced the model performance, which is clearly shown by the low calibration slope. The EARL2 dataset resulted in similar model performance and precision, but calibration slope indicated overfitting.

**Conclusion:**

In case of small datasets, it is not advisable to use a holdout or a very small external dataset with similar characteristics. A single small testing dataset suffers from a large uncertainty. Therefore, repeated CV using the full training dataset is preferred instead. Our simulations also demonstrated that it is important to consider the impact of differences in patient population between training and test data, which may ask for adjustment or stratification of relevant variables.

## Background

With technology advancements around biomarker development, the potential to generate multiple biomarkers for a single patient is expanding. In recent years, the potential of quantitative radiomics features derived from baseline ^18^F-FDG PET/CT scans to develop prognostic or predictive models is being explored. Several oncological studies have shown the added value of radiomics features when predicting outcome [1–6]. Often, radiomics analyses is performed with machine learning. Both machine learning and radiomics require large datasets. However, PET studies typically have small sample sizes. Consequently, many studies end up with multiple potential predictors using datasets with relatively small numbers of cases, leading to optimistic models that are adjusted to the data and decreased generalizability of the predictive model.

Optimism, or overfitting, is a well-known problem of predictive models: the model performance in new patients is often worse than expected from the development data set [7, 8]. The extent of optimism of pre-specified models can be estimated for similar patient populations using internal validation techniques such as bootstrapping [8], cross-validation or split-sample approaches [9]. With a split-sample approach you select part of your sample as a holdout set for validation; this sample is usually smaller than the training set. The latter frequently results in models with suboptimal performance due to small sample size. Split sample approaches can be used in very large samples but are not necessary because overfitting is no issue in this context [10].

External validation of a model is generally required to ensure that model prediction remain true in various settings [8, 10]. For clinical application this external validation is most interesting. Typically, external validity or generalizability is studied in an independent sample with patients from a plausibly related population. A split-sample (holdout) approach is often used when an external validation dataset is not available. However, this split-sample approach does not lead to truly external validation.

With the rise of many new biomarkers, scientific journals stress the need for the classical paradigm of external validation for new biomarkers. However, this might not always be feasible and methods traditionally used for internal validation are than the best alternative. In this study, we used simulated data to evaluate three internal validation methods: cross-validation (CV), holdout or bootstrapping against external validation using another external data set. Moreover, we tested the influence of the size of external test sets and different patient characteristics on validation performance.

## Methods

### Data

We simulated Ann Arbor stage-specific PET parameters and clinical characteristics for 500 patients, which is representative of a large PET dataset. Simulation parameters were generated based on data of 296 DLBCL patients from the HOVON-84 trial [5, 11]. Table 1 shows the PET and clinical parameters that were applied. For age > 60 years and World Health Organization (WHO) performance status > 1 fixed percentages were used that were observed clinically, whereas for log SUV_peak_, log MTV, log Dmax_bulk_ values were simulated using the mean and standard deviations (SD) of the values that were observed clinically. The Pearson correlations between individual PET parameters and clinical parameters were low (Pearson’s *r:* 0.05–0.52). Moreover, after log transformation PET features had normal distributions. As both assumptions for using random sampling were met (e.g., normal distribution and independent variables), the 500 patients were simulated using the *rnorm* function in R using the mean and SD of the log transformed PET parameters and frequencies of clinical parameters.

**Table 1:** PET and clinical characteristics used for simulations

Stage	Number of patients (%)	Log-SUV_peak_	Log-MTV	Log-Dmax_bulk_	Prevalence of age > 60 years (%)	Prevalence of WHO > 1 (%)
2	16	2.78 ± 0.50	12.0 ± 1.5	4.74 ± 0.6	30	23
3	21	2.78 ± 0.50	12.0 ± 1.5	5.4 ± 0.6	30	16
4	63	2.84 ± 0.50	13.0 ± 1.5	5.8 ± 0.6	30	6

### Calculation of probability

The probability (*p*) of progression within 2 years was calculated using the regression coefficients of our previously published model [5] using 296 patients from the HOVON-84 study.

1 $$p = \frac{1}{{1 + e^{{ - 6.532 + (0.533*\log \left( {{\text{MTV}}} \right)) - \left( {1.395*\log ({\text{SUV}}_{{{\text{peak}}}} )} \right) + \left( {0.257*\log \left( {D\max_{{{\text{bulk}}}} } \right)} \right) + \left( {0.773*{\text{IPIage}}} \right) + \left( {0.787*{\text{WHO}}} \right)}} }}$$

In that dataset, the cut-off for the probability for the high-risk group was 0.375. Thus all probabilities of 0.375 and higher were classified as having high risk for progression within 2 years, which resulted in a false positive rate (FPR) of 45% and a false negative rate (FNR) of 15%. In our simulation study, we used a probability of 0.375 as cut-off for high-risk patients, and randomly relabeled 45% of the high-risk patients as non-events and 15% of the low-risk patients as events to obtain the same FPR and FNR as seen in the actual clinical data.

### Statistical analyses

We analyzed model performance in terms of discrimination and calibration. Discrimination was expressed as the area under the curve (AUC) of the receiver-operating characteristic curve, the standard deviation of the AUCs for each fold and the confidence interval of AUCs over all folds and calibration was assessed using the calibration slope. The slope is 1 in a perfectly calibrated model. A calibration slope smaller than 1 indicates that predictions were too extreme (caused by overfitting of the model), leading to too low predictions for low risk patients and too high predictions for high risk patients. A calibration slope larger than 1 indicates that the spread in prediction is limited, with underestimation of high risk predictions and overestimation of low risk patients.

To assess internal model validity, we tested the influence of cross-validation, split-sample datasets and bootstrapping on model performance. To assess external model validity, we simulated external datasets with different sample sizes. Moreover, we simulated external datasets with different PET or patient characteristics to assess generalizability of the model. External datasets were simulated using the same approach as for the 500 simulated patients described under data. For the test set within the cross-validations and holdout set, patients were selected using the *sample* function in *R* stratified for Ann Arbor stage. For all external model validity approaches, we trained a model using fivefold 100 times repeated cross-validation using 400 simulated patients and then applied it to the simulated external dataset. For all internal and external validation approaches, the calculation of probability of progression was performed using Eq. 1. Moreover, the entire procedure was repeated 100 times by randomly reshuffling the data, resulting in a mean cross-validated AUC (CV-AUC), standard deviations (SD) and 95% confidence intervals (CI). Within each repeat, we determined overfitting in the regression coefficients of the best model by applying the train linear predictor (calibration slope) in the test datasets.

#### Internal model validity approaches

1. Fivefold repeated cross-validation using all 500 simulated patients. In this simulation 400 simulated patients were used for training and 100 for testing.
2. Fivefold repeated cross-validation using 400 simulated patients, the model was then applied on a holdout test set of 100 patients. This holdout set was not seen during training and had the same patient characteristics and PET metric distributions as the training set.
3. Bootstrapping as described by Harrell et al. [7]. Five hundred bootstrap samples were generated by resampling with replacement using all simulated patients.

#### External model validity approaches

4. A simulated external dataset of 100, 200 and 500 patients, respectively. The external test datasets were simulated using same patient characteristics and PET metric distributions.
5. A simulated external dataset of 500 patients where PET metrics were changed using offset and scale to mimic EARL2 reconstructed data. These reconstructions were based on non-small cell lung cancer and lymphoma patients with both EARL1 and EARL2 reconstructions [12].
6. A simulated external dataset of 500 patients where the prevalence of Ann Arbor stages 2,3 and 4 are changed from 16%, 21% and 63% to 33%,33%,34% and to 100% stage 2, 100% stage 3 and 100% stage 4, respectively.
7. A simulated external dataset of 500 patients where the threshold of the probability for high-risk patients was lowered to 0.10 and increased to 0.66 and 0.90, respectively.
8. A simulated external dataset of 500 patients where the FPR and FNR were changed both to 12%, both to 45% and both to 25%.

## Results

### Internal model validity

The apparent AUC of the model that included all 500 simulated patients was 0.73 (Table 2). The fivefold cross-validation using all simulated patients yielded a CV-AUC and 95% CI of 0.71 ± 0.06 (approach 1). The mean CV-AUC for the holdout set was 0.70 ± 0.07 (approach 2); both approaches had similar confidence intervals. The model performance using a bootstrap approach was slightly lower (approach 3; 0.67 ± 0.02) but model performance was more stable with a narrow confidence interval. As expected, the models suffered from overfitting. The calibration slope of all internal validation approaches was below 1 (range: 0.89–0.93), indicating that the coefficients of our model required shrinking.

**Table 2:** model performance expressed as CV-AUC and confidence interval and the standard deviation of models

Model	Discrimination: CV-AUC (± SD and 95% CI)	Calibration slope ± SD
Simulated true expected AUC	0.73	1
*Internal:*		
1: CV-AUC	0.71 ± 0.06 (0.59–0.81)	0.93 ± 0.41
2: 20% holdout test	0.70 ± 0.07 (0.57–0.82)	0.89 ± 0.33
3: Bootstrap	0.67 ± 0.02 (0.62–0.71)	0.90 ± 0.37
*External validation*		
4: External test		
*n* = 100	0.69 ± 0.07 (0.56–0.83)	1.05 ± 0.45
*n* = 200	0.70 ± 0.05 (0.61–0.80)	1.01 ± 0.23
*n* = 500	0.70 ± 0.03 (0.64–0.74)	1.04 ± 0.17
5: EARL 2	0.70 ± 0.07 (0.56–0.82)	0.83 ± 0.33
6: Prevalence stage		
33–33–34	0.66 ± 0.04 (0.59–0.74)	0.99 ± 0.21
100% 2	0.59 ± 0.04 (0.49–0.66)	0.41 ± 0.14
100% 3	0.63 ± 0.04 (0.56–0.71)	0.56 ± 0.12
100% 4	0.72 ± 0.03 (0.66–0.76)	1.56 ± 0.28
7: Positivity rate		
0.10	0.73 ± 0.02 (0.69–0.78)	1.07 ± 0.15
0.66	0.64 ± 0.04 (0.58–0.70)	0.32 ± 0.12
0.90	0.61 ± 0.04 (0.53–0.66)	0.25 ± 0.11
8: FPR/FNR		
0.12/0.12	0.78 ± 0.03 (0.73–0.83)	1.78 ± 0.22
0.45/0.45	0.53 ± 0.02 (0.49–0.57)	0.15 ± 0.10
0.25/0.25	0.66 ± 0.03 (0.61–0.71)	0.86 ± 0.10

*CV-AUC* cross-validated area under the curve, *SD* standard deviation, *CI* confidence interval, *FPR* false positive rate, *FNR* false negative rate

### External model validity

**Fig. 1:**
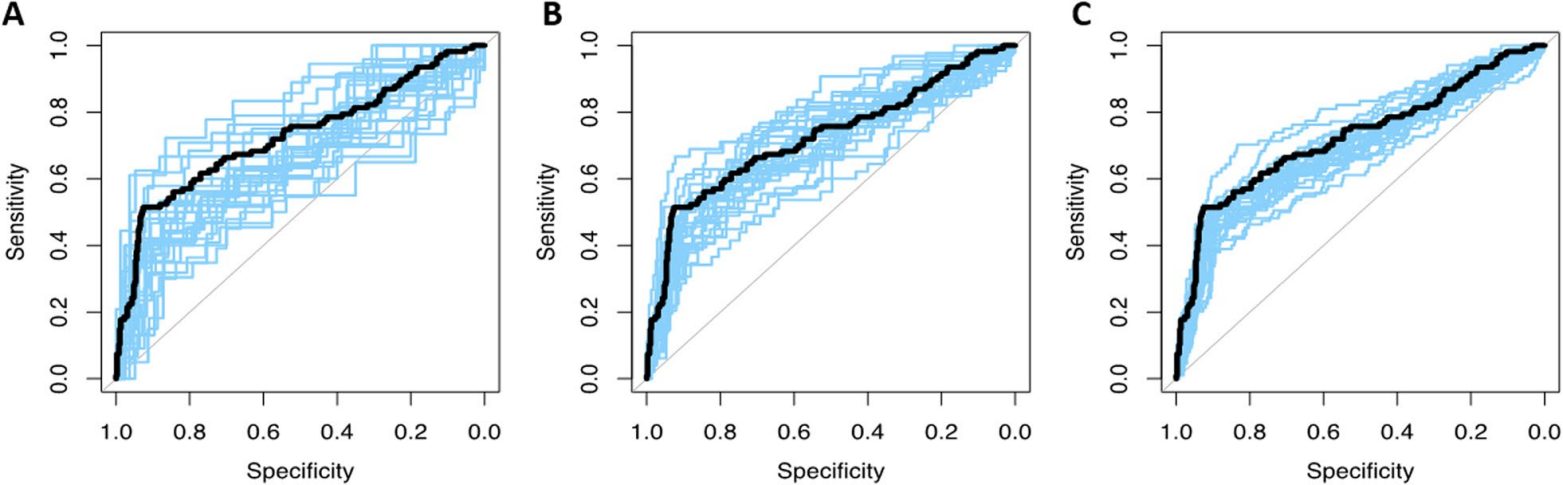
AUCs of 25 repeats of external simulated datasets with different sizes in blue with the simulated true expected AUC in black **A** an simulated external dataset of 100 patients, **B** simulated external dataset of 200 patients and **C** simulated external dataset of 500 patients

An external test set of 100 newly simulated patients resulted in a CV-AUC of 0.69 ± 0.07, 200 newly simulated patients resulted in a CV-AUC of 0.70 ± 0.05 and an external dataset of 500 newly simulated patients yielded a CV-AUC 0.70 ± 0.03 (approach 4; Fig. 1). With larger sample size of the externally simulated datasets the standard deviation decreased which as a consequence resulted in smaller confidence intervals. Because the patient characteristics and PET metric distributions were identical between training and external validation, the mean calibration slope is close to 1 indicating good calibration of the model coefficients. As the sample size increased, the calibration between the model coefficients was better, which is shown by the smaller standard deviations of the calibration slope (*n* = 100: 1.05 ± 0.45 vs 1.04 ± 0.17 for *n* = 500).

**Fig. 2:**
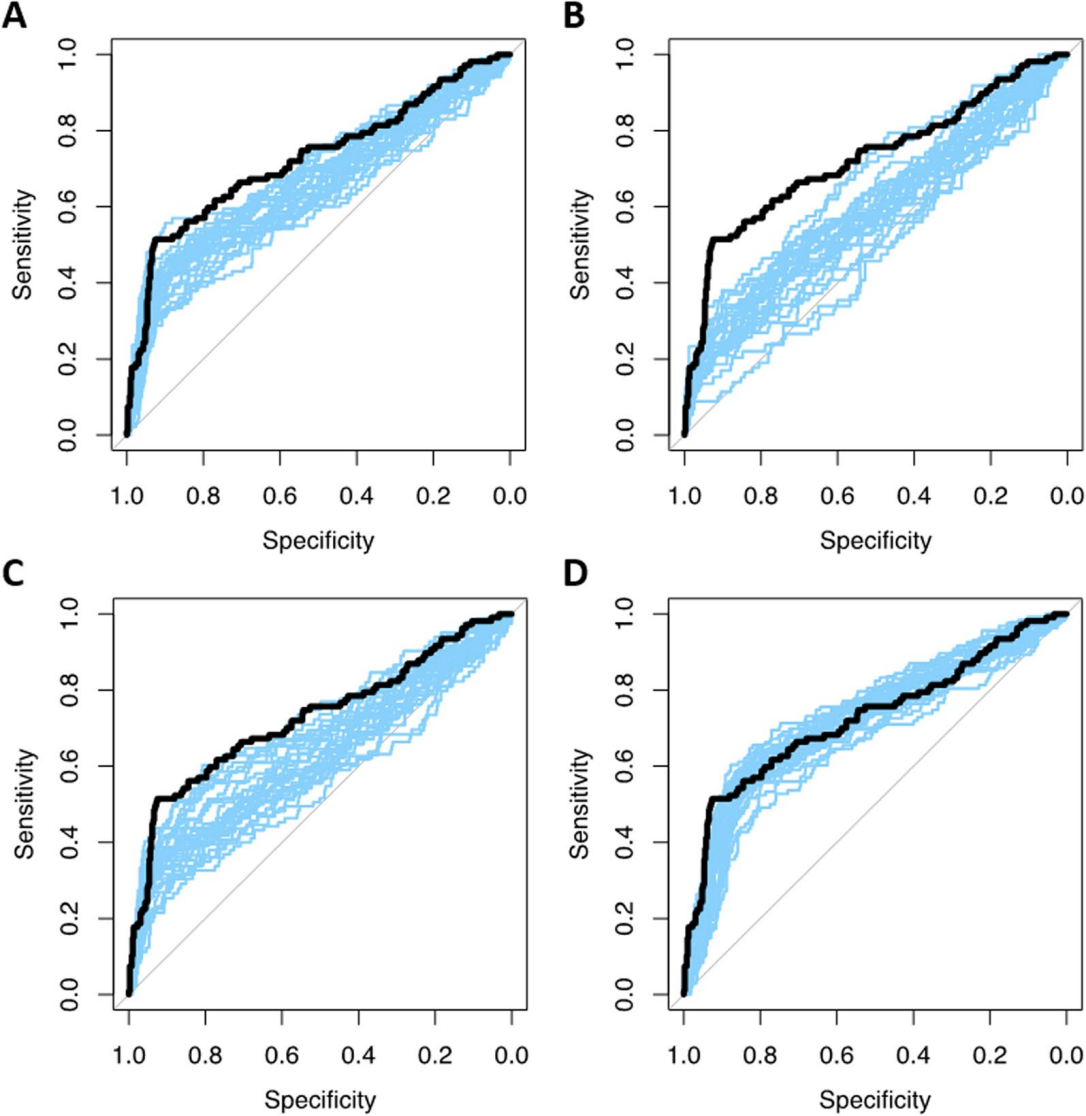
AUCs of 25 repeats of external simulated datasets with different distributions of Ann Arbor stage in blue with the simulated true expected AUC in black **A** simulated dataset with 33% Stage 2, 33% Stage 3 and 34% stage 4 patients, **B** simulated dataset with Stage 2 patients, **C** simulated dataset with Stage 3 patients, **D** Simulated dataset with stage 4 patients

**Fig. 3:**
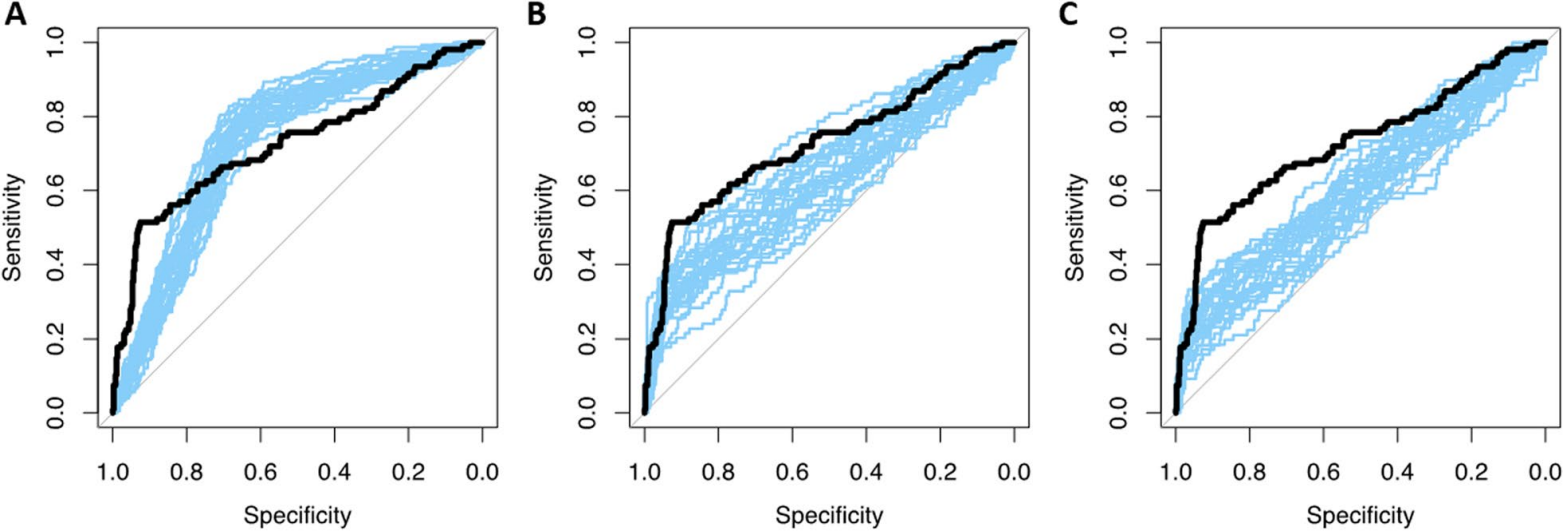
AUCs of 25 repeats of external simulated datasets varying the positivity rate in blue with the simulated true expected AUC in black **A** positivity rate of 0.10, **B** positivity rate of 0.66, **C** positivity rate of 0.90

When applying an offset and scaling to mimic an EARL2 reconstructed dataset, the model performance was 0.70 ± 0.07 (approach 5). The mean calibration slope of this model was 0.83, indicating overfitting of the model leading to too low predictions for low-risk patients and too high predictions for high-risk patients. If prevalence of all stages in the external test set was equal, the model yielded a CV-AUC of 0.66 ± 0.04. Model performance increased from 0.59 ± 0.04 when only Ann Arbor stage 2 patients were included to 0.72 ± 0.03 when only Ann Arbor stage 4 patients were included in the external test set (approach 6; Fig. 2). As expected, calibration of the models where only one Ann Arbor stage was included showed poor calibration. For Ann Arbor stage 2 and 3 models suffered from overfitting with calibration slopes of 0.41 ± 0.14 and 0.56 ± 0.12, respectively. Including only Ann Arbor stage 4 patients led to underestimation of high-risk predictions, as shown by the calibration of 1.56 ± 0.28. Lowering the positivity rate to 0.10 resulted in slightly higher model performance (approach 7; CV-AUC 0.73 ± 0.02) and slightly decreased spread in predictions causing underestimation of high-risk and overestimation of low-risk patients (calibration slope: 1.07 ± 0.15). Higher cut-offs resulted in lower model performance (approach 8; CV-AUC 0.64 ± 0.04 and 0.61 ± 0.04; Fig. 3), with lower model calibration causing overfitting of the model coefficients with extreme predictions (calibration slope 0.32 ± 0.12 and 0.25 ± 0.11, respectively). As expected, a FPR and FNR of 12% yielded in a higher CV-AUC (0.78 ± 0.03), and a FPR and FNR of 45% in lower model performance (approach 9; CV-AUC 0.53 ± 0.02). A FPR and FNR of 25% resulted in lower results compared to our original model (CV-AUC 0.66 ± 0.03) with poor model calibrations.

## Discussion

This study showed that in case of small sample sizes, there is no difference in performance when using a holdout approach (approach 2) or a small external dataset (approach 4, *n* = 100) with similar patient characteristics and image qualities. A single small dataset suffers from large uncertainty suggesting that repeated cross-validation using the full dataset is preferred instead in this situation. Moreover, external validation has limited additional value when patient and PET characteristics are similar (approach 4). However, our simulations demonstrated that external datasets with different patient or PET characteristics have added value, and these differences may ask for adjustment of relevant variables in the final model (approaches 5–9), as shown by the calibration slopes of the models. Yet again, small datasets result in large uncertainty in model performance.

In line with other studies [9, 13], our simulation results show that no single internal validation method clearly outperformed other internal validation methods when looking at the CV-AUCs, standard deviation and calibration slopes (approaches 1–3). Bootstrapping (approach 3) resulted in a smaller standard deviation than cross-validation or holdout. Model performance using a bootstrap approach resulted in slightly lower but more stable model performance. Although mean calibration slopes and mean CV-AUC are comparable for all internal validation approaches, large differences in model performance are observed per fold, stressing the need for repeated validation.

Using a small training set or test set may not be representative of all the possible cases. A small training set results in poor generalization ability and a small test set leading to large confidence intervals. This is also shown in our simulation results, where the confidence interval became smaller as the sample size of the external test set increased (Approach 4). Similarly, the uncertainty of the predictions was lower in the cross-validated model where all simulated patients were used, compared to the uncertainty in the holdout approach, thereby reducing the sample size for the cross-validation training. Moreover, using a holdout set as test set is essentially the same as onefold of the cross-validation as the patient characteristics and metric distributions are identical for the training and test set for both the cross-validation and holdout approaches. Therefore, a holdout set is only effective if you have a very large dataset [10]. As PET studies often have small sample sizes (< 100 patients) a CV-AUC or bootstrap approach is favored over a holdout set in small datasets. Moreover, larger external datasets with similar patient and PET characteristics only result in higher certainty of model predictions as shown by lower standard deviations in CV-AUC and calibration slope, but do not provide meaningful information about generalizability.

The focus of a validation study should not be on the statistical testing of differences in performance but on generalizability of the model in other settings [8, 10]. Our study showed that PET and patient characteristics, such as EARL reconstruction and Ann Arbor stage, influence the model validation (approaches 5–6), this effect is more prominent in the lower calibrations between the models. A model with high generalizability is more likely to be implemented in clinical practice. Often, an external dataset is not available and a training set that is not representative (e.g., due to aberrant patient or PET characteristics) might lead to overfitting of the model in the training set, reducing its performance in the test set and therefore reducing its clinical applicability. Therefore, it is important to check the influence of patient and PET characteristics within your sample using simulations, if possible. An external dataset allows to assess case-mix differences, whereas internal validity approaches only correct for sampling variation.

It is important to note that for this simulation study we assumed that the model to predict outcome was fixed and the test set is only used to validate the model that was developed in the training set. Therefore, our results only apply for the validation of a fixed model. If a new model was trained or feature selection was incorporated in the training set a holdout set or external set would not be comparable to a cross-validation approach. However, in this scenario a small validation set also results in large uncertainty of model performance. Moreover, a holdout set always results in a smaller training set, thereby leading to larger uncertainty for both the training and validation set. Therefore, most of our conclusions remain the same when selecting models and/or features in your training set. However, feature selection leads to overfitting of the training set.

Based on our results we can conclude that in case of small sample sizes there is no added value of a holdout approach (internal validation) or a very small external dataset with similar patient and PET characteristics. PET studies often have small sample sizes; therefore, a holdout approach is not favored as it leads to larger uncertainties for both the training set and validation set. Moreover, a single small external dataset also suffers from a large uncertainty. External validation provides important information regarding the generalizability of a model in different settings. Our simulations also demonstrated that it is important to consider the impact of differences in patient population between training, cross-validation and (external) testing data, which may ask for adjustment or stratification of relevant variables or recalibration of the models. Therefore, we suggest that for future studies with small sample sizes, a repeated CV or bootstrap approach is superior to holdout or only one small external test set with similar patient characteristics, and editors should stress the need for proper internal validation of models.

## Data Availability

Simulated data and scripts are available upon reasonable request.
